# Aspirin‐induced urticaria in a recently diagnosed ischemic stroke patient: A case report and literature review

**DOI:** 10.1002/ccr3.7704

**Published:** 2023-08-07

**Authors:** Abhinav Dahal, Sushant Gautam, Aliza Shakya, Ashmita Pant, Kriti Bhandari, Shumneva Shrestha, Sajina Shrestha, Abhigan Babu Shrestha

**Affiliations:** ^1^ Sukraraj Tropical and Infectious Disease Hospital Kathmandu Nepal; ^2^ Nepalgunj medical college Banke Nepal; ^3^ Manipal college of medical sciences Pokhara Nepal; ^4^ Nepal Medical College Kathmandu Nepal; ^5^ Maharajgunj Medical Campus, Institute of Medicine, Tribhuvan University Kathmandu Nepal; ^6^ Kist Medical College Patan Nepal; ^7^ M Abdur Rahim Medical College Dinajpur Bangladesh

**Keywords:** aspirin, hemiplegia, stroke, urticarial

## Abstract

**Key Clinical Message:**

NSAIDs may be rare but an important cause of urticarial which should not be missed.

**Abstract:**

The aspirin and urticaria correlation has not been fully understood. The pharmacological inference is suspected to be the diversion of arachidonic acid metabolism. Aspirin sensitivity can aggravate preexisting chronic urticaria and in some instances causes acute urticaria. We report a case of a 53‐year‐old male, recently diagnosed with a stroke, who presented with complaints of multiple rashes over the trunk and upper extremities with aspirin. NSAIDs induced urticarial are usually neglected by physicians during diagnosis.

## 1 INTRODUCTION

Aspirin is a drug that is widely used to reduce the risk of stroke, transient ischemic attack, and cardiovascular events. This is due to its antithrombotic properties by irreversibly inhibiting the cyclooxygenase enzyme, reducing the synthesis of thromboxane A2 and thus subsequently reducing platelet aggregation.^1^, ^2^, ^3^ Aspirin and NSAIDs' pharmacological properties also result in adverse reactions like GI upset, renal toxicity, and hemorrhagic complications, as well as potentiate hypersensitivity reactions.^4^ Aspirin hypersensitivity is reported by 0.9%–1.5% of the general population.^5^ Hypersensitivity reactions to NSAIDs are classified based on their involvement of the skin, like urticaria or angioedema, the airways or other organs, their acute or delayed onset, the presence of underlying diseases, and their cross reactivity.^6^ In this report, we document a case of aspirin‐induced urticaria in a patient with a recent history of ischemic stroke and hypertension.

## 2 CASE DESCRIPTION

**FIGURE 1:**
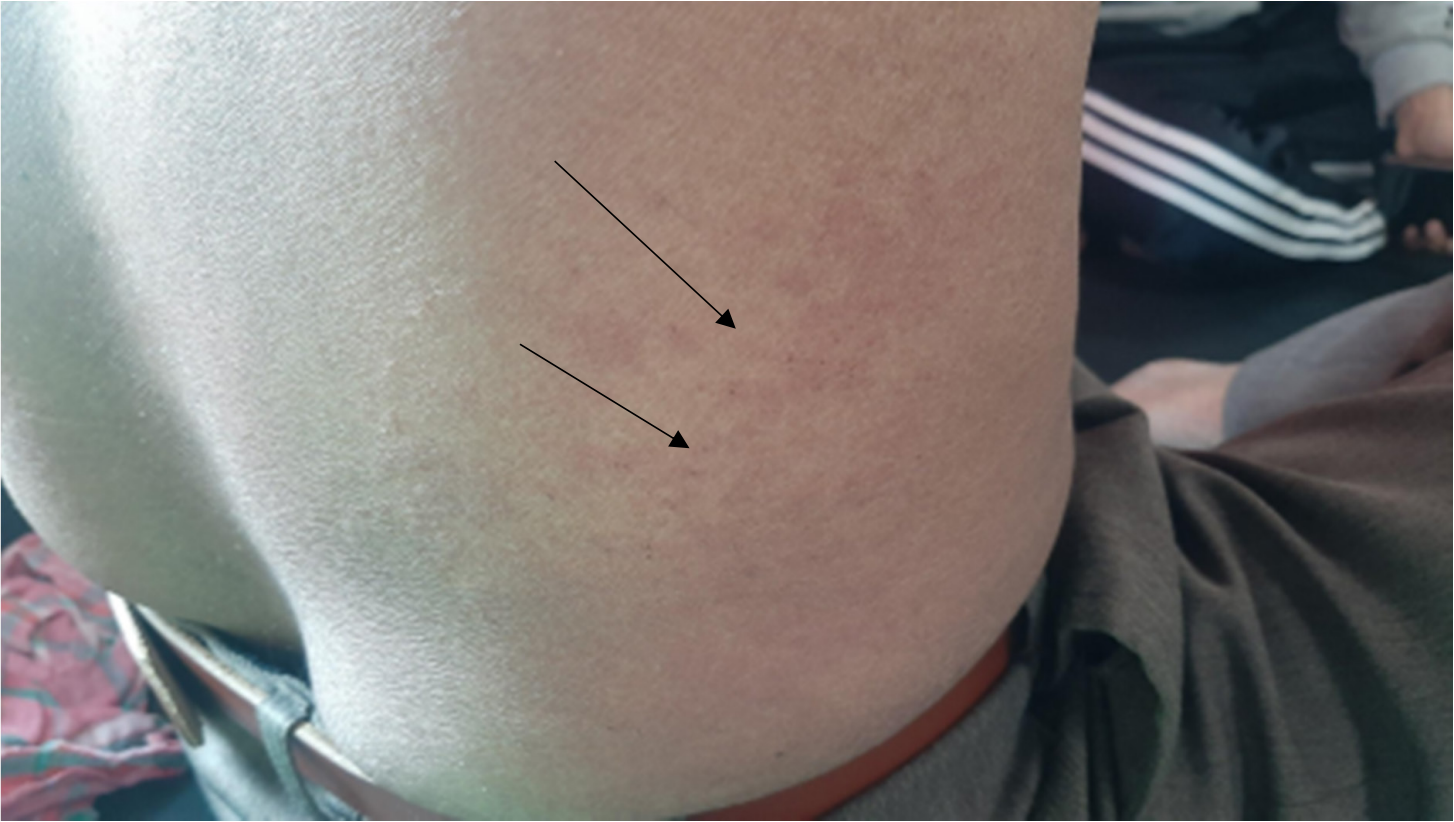
Multiple pruritic erythematous macules over lower part of trunk.

**FIGURE 2:**
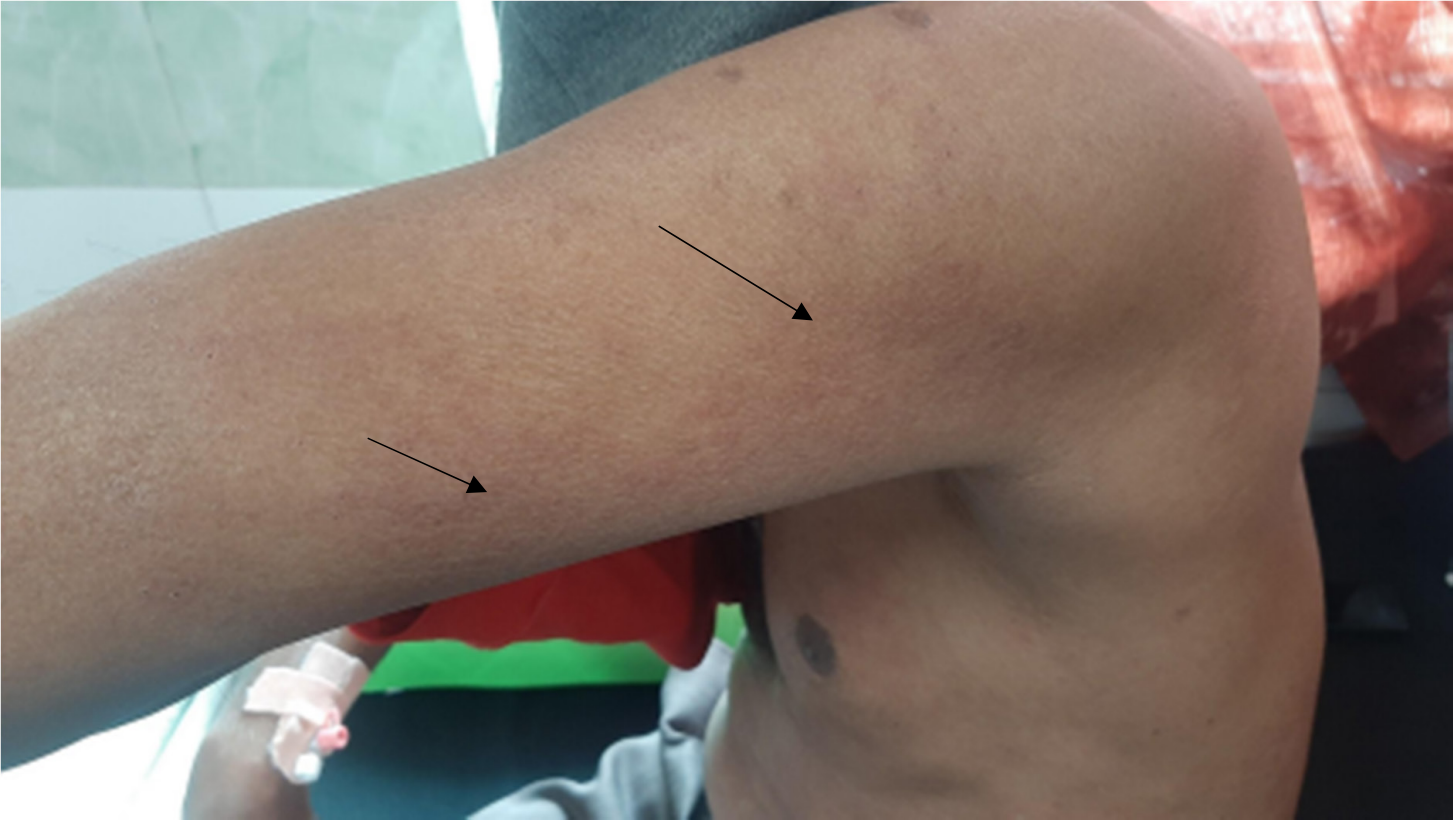
Multiple pruritic erythematous macules over upper limb.

A 53‐year‐old male presented to the emergency department with a chief complaint of multiple rashes with a burning and itching sensation over the trunk and extremities. Figures 1 and 2.

The patient was a known case of hypertension with a recent diagnosis of left‐sided ischemic stroke with right‐sided hemiparesis 7 days back for which he was admitted for 3 days. There were no significant reactions to the treatment noticed during the hospital stay. He was discharged with a daily dose of aspirin 75 mg o.d., enalapril 10 mg o.d., atorvastatin 20 mg o.d., and omeprazole 20 mg b.d. He was advised for proper bed rest with two hourly posture changes and physiotherapy.

He was solely on prescribed medications for the past 4 days. On the next day, he was admitted following the appearance of cutaneous manifestations with no other associated symptoms such as angioedema or shortness of breath. He had no prior history of allergic reactions. On further inquiry, he denied any changes to diet or environmental stimuli including no exposure to any pets or animals. He also gave a negative history of family members having such allergic manifestations.

On examination, multiple, pruritic, erythematous, and blanching macules were present on bilateral upper extremities and trunk (Figure 1). His vital signs were within normal limits. Leukocytosis (wbc:12,000 cells/mm^3^) with higher eosinophil count (550 per mL of blood) was noted while other complete blood counts and comprehensive metabolic panels were unremarkable.

Medical history revealed he was taking enalapril 10 mg o.d. for the past 3 years for hypertension. As ACE inhibitors are commonly responsible for causing the side effects such as allergies, drug rash, and even angioedema, it was replaced with amlodipine 5 mg o.d. He was started on histacin 4 mg b.d for symptomatic management. However, there was no significant improvement in his condition. Therefore, we decided to stop the current medication one at a time to identify the culprit drug.

Aspirin was replaced with clopidogrel 75 mg o.d. and given daily. He was kept under observation and followed up the next day with improvement. The patient told that his itching and the degree of rash spread through his body were diminished. Three days later, the rashes almost fully subsided and he was advised to continue histacin for 1 more week without stopping clopidogrel intake. Aspirin was identified as the sole cause of the rashes. He was discharged following complete recovery of cutaneous symptoms after 3 days with proper counseling regarding his condition and precaution to be taken for future aspirin use.

## 3 DISCUSSION

Aspirin and other NSAIDs are known to cause hypersensitivity reactions. One of the manifestations of such reactions is urticaria. The prevalence of aspirin‐induced urticaria is estimated to be around 0.3% excluding individuals with recurrent urticaria, hay fever, and chronic chest disease.^7^ Aspirin‐induced urticaria(AIU) can be classified into two types aspirin‐intolerant acute urticaria(AIAU) and aspirin‐intolerant chronic urticaria(AICU). The symptoms develop within minutes to 24 h in AIAU and last for less than 6 weeks whereas in AICU symptoms typically last for more than 6 weeks.^8^

Elevated IgE levels and atopy are suggested as common predisposing factors for AIU according to a genetic study done in the Korean population.^8^ In addition, HLA‐DRB1*1302‐DQB1*0609 also has been identified as a genetic marker.^9^ Besides this, various genetic studies have reported that high‐affinity IgE receptors,^10^ histamine *N*‐methyltransferase,^11^ and adenosine A3 receptors^12^ are other genetic determinants for AIU. These findings allude that, causes leading to the release of inflammatory mediators either by increased histamine release, faulty histamine degradation, or augmented mast cell signaling, contribute to the manifestation of AIU.

**FIGURE 3:**
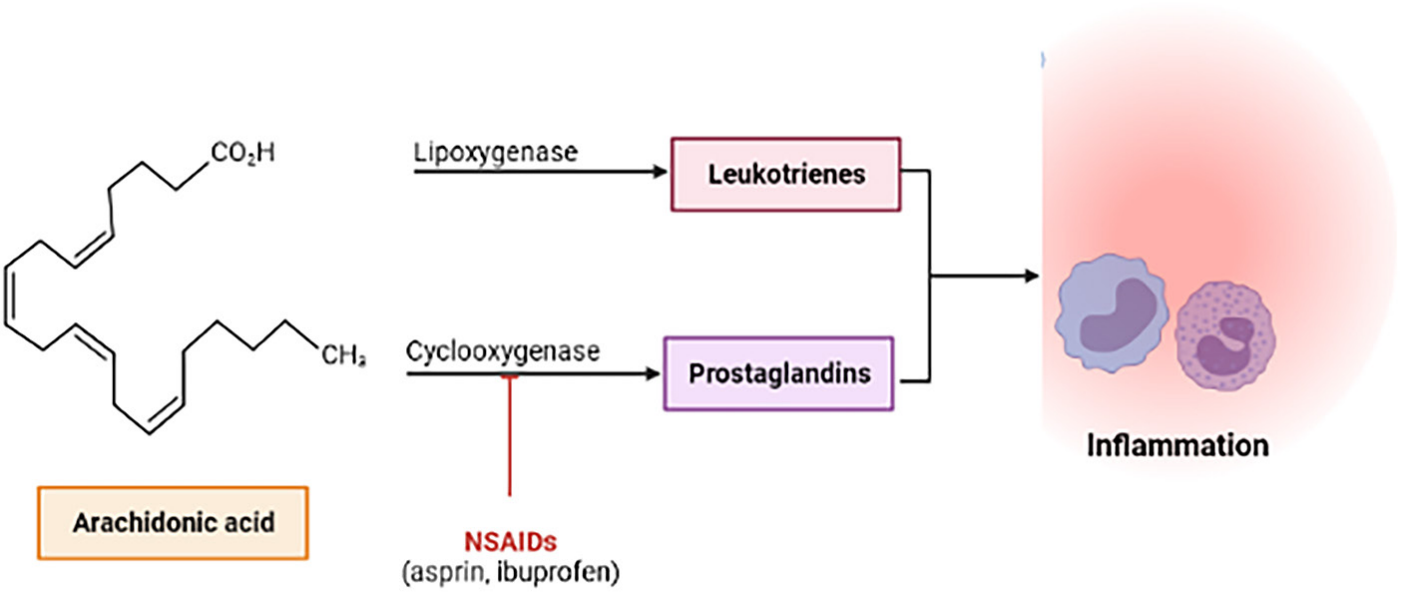
Mechanism of inflammation caused by aspirin (NSAIDs).

NSAIDs and aspirin are known to be the common drugs causing hypersensitivity drug reactions.^13^ Drug hypersensitivity reactions constitute about one‐third of all adverse drug reactions and affect 10%–20% of hospitalized patients and 7% of outpatients.^14^ Depending on the type of hypersensitivity reaction, the patient can develop an array of cutaneous manifestations ranging from urticaria/angioedema, fixed drug eruption, and maculopapular exanthem to more severe manifestations such as DRESS or SJS/TEN.^15^ We have previously encountered a case of Stevens–Johnson Syndrome, a Type III hypersensitivity reaction due to an antibiotic namely cefixime.^16^ In this case, we have encountered a case of single NSAID‐induced urticaria/angioedema or anaphylaxis, a Type I hypersensitivity reaction Figure 3.

To date, there are only a few case reports published in PubMed highlighting the association of aspirin with urticaria. This may be because aspirin sensitivity is often neglected because of the cross‐reaction with NSAIDs. One case report has documented urticaria in three patients caused by aspirin where pharmaceutical excipients present in the formulation of the drug were found to be the sole cause for the hypersensitivity reaction in two of those patients.^17^ However, acute urticaria is not uncommon and when patients present with an urticarial reaction clinicians do not consider NSAIDs as the cause, as the reaction occurs in the context of different triggers and is thus difficult to establish.

Beyond drug‐induced urticaria, differential diagnosis of acute urticaria includes urticaria attributed to infections, foodstuffs, contact dermatitis, solar urticaria, cholinergic urticaria, arthropod bite reactions, autoimmune disorders, and small vessel vasculitis. Diagnostic workup for many of these etiologies relies on laboratory parameters and histological evidence in addition to history. Initial laboratory investigations revealed leukocytosis with a predominance of eosinophils. However, access to further testing was limited due to the patient's refusal and unfortunately no blood investigations or skin biopsy was taken at presentation.

Management of NSAID‐induced urticaria includes strict culprit NSAID avoidance along with symptomatic management of acute urticaria.^18^ Antihistamines are the first‐line treatment for the management of acute urticaria. In severe cases, corticosteroids can be added to control symptoms.^19^, ^20^ Some types of NSAID hypersensitivity are known to exhibit cross‐reactivity thus strict NSAID avoidance should be done until the potential of cross‐intolerance is ruled out. If a patient has a history suggesting selective NSAID‐induced urticaria, the challenge to chemically unrelated strong COX‐1 inhibitor may be done to rule out crossreactivity type hypersensitivity.^6^ Once a diagnosis of SNIUAA is established, the use of a drug allergy passport and patient education help both the medical provider and the patient know about the NSAID hypersensitivity status, avoidance of the culprit NSAID as well as the use of chemically unrelated NSAIDs as safe alternatives. In this entity, an alternative drug to aspirin must be sought.^6^, ^18^ Therefore our clinical reasoning is composed of detailed patient history, physical examination and clinical course. Our patient did not have any history of multisystem involvement, recent infection, past medical history of atopy, inducible urticaria due to heat, cold, or stress, or recent contact with common irritants or insect bites.

The patient had been taking aspirin, enalapril and atorvastatin following the diagnosis of ischemic stroke with right‐sided hemiparesis. The urticaria and pruritus should not be caused by enalapril, as the patient was tolerating the medication well for the past 3 years and the patient's symptoms worsened even after withdrawing the drug. The temporal correlation between the appearance of urticarial rash and aspirin intake, and the resolution of urticarial rash and pruritus following aspirin discontinuation suggests the possibility of aspirin‐induced urticaria. The causality assessment of the adverse drug reaction (ADR) was carried out using the Naranjo scale, a method for estimating the probability of adverse drug reactions. The assessment revealed the ADR to be “Probable” (+6) to be associated with aspirin.^21^ The patient's history notably lacked the presence of chronic urticaria. While the drug provocation test for cross intolerance was not done, the patient's history reveals previous tolerance to other NSAIDs like ibuprofen and paracetamol.

## 4 CONCLUSION

In conclusion, our case highlights the importance of considering aspirin hypersensitivity as a potential cause of cutaneous symptoms in patients with underlying medical conditions, particularly stroke. Although aspirin is generally well‐tolerated, it can cause adverse reactions such as urticaria. Clinicians should be aware of this possibility and appropriately diagnose, manage, and counsel patients to prevent future adverse reactions. The treatment for aspirin‐induced urticaria involves discontinuing aspirin and prescribing alternative medications such as clopidogrel, along with counter medications like corticosteroids, antihistamines, and immunomodulators in severe cases.

## AUTHOR CONTRIBUTIONS

**Abhinav Dahal:** Writing – original draft; writing – review and editing. **Sushant Gautam:** Writing – original draft; writing – review and editing. **Aliza Shakya:** Writing – original draft; writing – review and editing. **Ashmita Pant:** Visualization; writing – original draft. **Kriti Bhandari:** Writing – original draft; writing – review and editing. **Shumneva Shrestha:** Writing – review and editing. **Sajina Shrestha:** Writing – review and editing. **Abhigan Babu Shrestha:** Supervision; writing – review and editing.

## FUNDING INFORMATION

None.

## CONFLICT OF INTEREST STATEMENT

There are no conflicts of interest.

## ETHICS STATEMENT

N/A.

## CONSENT

Written informed consent was obtained from the patient for the publication of this case report and accompanying images. A copy of the written consent is available for review by the Editor‐in‐Chief of this journal on request.

## Data Availability

All the data are available in the manuscript.
